# Prevalence, Clinical and Virologic Outcomes of Hepatitis B Virus Co-Infection in HIV-1 Positive Kenyan Women on Antiretroviral Therapy

**DOI:** 10.1371/journal.pone.0059346

**Published:** 2013-03-18

**Authors:** Summer L. Day, Katherine Odem-Davis, Kishorchandra N. Mandaliya, Keith R. Jerome, Linda Cook, Linnet N. Masese, John Scott, H. Nina Kim, Susan M. Graham, R. Scott McClelland

**Affiliations:** 1 Department of Medicine, University of Washington, Seattle, Washington, United States of America; 2 Department of Epidemiology, University of Washington, Seattle, Washington, United States of America; 3 Fred Hutchison Cancer Research Center, Seattle, Washington, United States of America; 4 Pathcare Laboratories, Mombasa, Kenya; 5 Department of Laboratory Medicine, University of Washington, Seattle, Washington, United States of America; 6 Institute of Tropical and Infectious Diseases, University of Nairobi, Nairobi, Kenya; 7 Department of Global Health, University of Washington, Seattle, Washington, United States of America; Alberta Provincial Laboratory for Public Health/University of Alberta, Canada

## Abstract

**Background:**

Sub-Saharan Africa carries a high burden of co-infection with HIV-1 and hepatitis B virus (HBV). In this region, individuals with HIV-1/HBV co-infection on antiretroviral therapy (ART) frequently receive lamivudine as the only agent active against HBV, raising concerns for development of HBV resistance to lamivudine. We aimed to determine the prevalence, clinical, and virologic outcomes of chronic HBV infection, including HBV resistance to lamivudine, in a cohort of HIV-1 seropositive Kenyan women on long-term ART.

**Methods:**

In this prospective cohort study, HIV-1 seropositive women initiated three-drug ART regimens that included lamivudine as the single drug active against HBV. Archived samples were tested for HBsAg, with further testing to determine HBeAg seroprevalence, HBV DNA suppression, and lamivudine resistance. We estimated the prevalence of chronic HBV and examined associations between HBV co-infection and clinical and virologic outcomes with chi-square tests, logistic regression, Kaplan-Meier and Cox regression.

**Results:**

In a cohort of 159 women followed for a median of 3.4 years (interquartile range 1.4–4.5), 11 (6.9%; 95% CI 3.1–10.7) had chronic HBV infection. Of these, 9 (82%) achieved undetectable plasma HBV DNA levels. One woman developed lamivudine resistance, for an incidence of 3 per 100 person-years. The HBV co-infected women were at greater risk for abnormal ALT elevations compared to HIV-1 mono-infected women (HR 2.37; 95% CI 1.1–5.3). There were no differences between HBV-infected and uninfected women in mortality, CD4 count, or HIV-1 RNA suppression.

**Conclusion:**

The prevalence of chronic HBV in this cohort was similar to recent studies from other African populations. Given our long-term follow-up, lamivudine resistance was lower than expected for HIV-1/HBV co-infected patients. Improved screening for HBV and extended follow-up of HIV-1/HBV co-infected individuals are needed to better understand the impact of different ART regimens on clinical outcomes in this population.

## Introduction

In sub-Saharan Africa, 8–20% of people with HIV-1 infection are estimated to be co-infected with hepatitis B virus (HBV) [1]. In areas of high endemicity, such as sub-Saharan Africa, most individuals are infected with HBV in early childhood (due to close contact with household members) or in the perinatal period (from mother to baby at birth). In fact, it is estimated that 70–90% of individuals in the region have previously been exposed to or are chronic carriers of HBV [2]. This is in contrast to areas of low endemicity, such as in western countries, where most transmission occurs in adolescence and young adulthood due to high risk behaviors (unprotected sexual contact and injection drug use) [3].

Compared to those with HBV infection alone, HIV-1/HBV co-infected individuals have higher HBV viral loads, decreased hepatitis B e antigen (HBeAg) seroconversion, and increased rates of liver disease [2], [4], [5]. Co-infection with HBV may also influence the response to antiretroviral therapy, including HIV-1 RNA suppression and increase in CD4 count [6]. The increased availability of antiretroviral therapy (ART) in resource-limited settings has improved survival of individuals with HIV-1 [6]. As life expectancy improves, HBV can be expected to emerge as an important cause of morbidity and mortality in HIV-1 infected individuals on ART [2], [7].

Beginning in 2002, World Health Organization (WHO) guidelines for first-line ART included lamivudine as the only agent active against HBV [8]. In the setting of lamivudine monotherapy, HBV resistance has been observed at rates of 20% per year in Western cohorts [9]. As of 2010, WHO guidelines recommend a first-line regimen including tenofovir and either lamivudine or emtricitabine in HIV-1/HBV co-infected patients [10], [11]. However, in resource-limited settings HBV screening is limited, HBV status is often unknown, and tenofovir may still be unavailable [12],[13]. As a result, many ART regimens in sub-Saharan Africa continue to include lamivudine as the only agent active against HBV [12]–[14].

Compared to studies in Western cohorts, studies of HIV-1/HBV co-infected individuals in sub-Saharan Africa taking lamivudine-containing ART have suggested lower rates of HBV resistance to lamivudine. A cross-sectional study in Cameroon found resistance mutations in 7 (13%) of 54 patients at 24 months [12]. A retrospective analysis from The Gambia found lamivudine resistance mutations in 3 (14%) of 21 patients followed between 6 and 56 months [15]. Even lower levels of resistance were found in a recent prospective cohort study from Nairobi, Kenya, which reported that only 2 (9.5%) of 27 co-infected patients developed lamivudine resistance after 18 months of therapy [16].

Few studies in either Western or African nations have followed HIV-1/HBV co-infected patients for periods longer than 24 months. This is a concern, since the risk of resistance mutations increases with time on lamivudine [9], [13], and ART is expected to be life-long. To address limitations in the available literature, we conducted a prospective cohort study over 6 years to determine the prevalence, clinical, and virologic outcomes of chronic HBV infection, including HBV resistance to lamivudine, in HIV-1 seropositive Kenyan women on long-term ART.

## Methods

This prospective cohort study included HIV-1-seropositive, non-pregnant women on long-term ART. These women were part of an open cohort study established in 1993 to identify risk factors for HIV-1 acquisition in female sex workers in Mombasa, Kenya. Detailed methods for this cohort have been published previously [17], [18]. Briefly, ART was offered to women eligible for treatment according to Kenyan Guidelines (CD4 cell count <200 cells/mL or AIDS-defining illness) from March 2004 through March 2010. All participants started ART containing lamivudine with nevirapine and either zidovudine or stavudine. Monthly follow-up visits included a standardized interview and physical examination according to previously published methods [17]. Blood was collected at baseline, and every three months thereafter, for monitoring of CD4 count and alanine aminotransferase (ALT) levels (Reflotron, Roche, Indianapolis, IN). Adherence was monitored at each visit by pill count ([number of pills taken divided by total number expected] x100%). If patients failed to bring their medication containers to follow-up visits, adherence was calculated on the basis of patient recall.

Plasma samples were frozen at −70°C until shipment on dry ice to Seattle for HIV-1 viral load [17] and HBV assays [16]. Baseline samples were tested for hepatitis B surface antigen (HBsAg) and core antibody (HBcAb) with an ADVIA Centaur chemiluminometric immunoassay system (Siemens, Tarrytown, NY). Chronic HBV carriers were defined as HBsAg positive. All chronic HBV carriers underwent quantification of HBV DNA at baseline and quarterly thereafter. They were also tested for HBeAg and antibody using an enzyme-linked immunoassay. If HBeAg was positive, subsequent samples were tested for HBeAg seroconversion at the time of HBV DNA suppression and at the end of follow-up. HBV genotype and lamivudine resistance mutations were investigated by sequencing the YMDD region of the HBV polymerase gene at baseline and future time points if HBV DNA remained detectable (≥100 copies/ml) after three months.

This analysis used the intent-to-treat principle, which included women who discontinued ART or had poor adherence. Prevalence of HBV was estimated with 95% confidence intervals (CIs) by bootstrap re-sampling. Chi-square tests were used to assess correlates of HBV infection including age, education level, alcohol use, and reported sexual risk behaviors (unprotected sex, abstinence, 100% condom use, >1 sex partners per week, >2 sex acts per week, age at first sex, and years in transactional sex). Associations of chronic HBV infection with mortality, time to CD4 lymphocyte count >350, and time to first ALT elevation were assessed by Kaplan-Meier and Cox regression. Associations with HIV-1 RNA suppression at six months and one year were examined by logistic regression. Analyses were conducted using SPSS version 18.0 (SPSS Inc., Chicago, IL) and R version 2.13 (The R Foundation, ISBN 3-900051-07-0).

### Ethics Statement

Ethical review committees of the Kenya Medical Research Institute and the University of Washington approved this study. All participants provided written informed consent.

## Results

This analysis included 159 HIV-1 seropositive, non-pregnant women who initiated ART and had baseline samples available for HBV testing. These women contributed 479 person-years of follow-up (median 41 months, interquartile range [IQR] 17–54 months). Twenty-seven (21%) participants were lost to follow-up (defined as greater than 60 days elapsed from previous visit at the end of the study). Seven (4.4%) women died, all of whom were HBsAg negative.

At baseline, participants’ median age was 37 (IQR 33–41) years. Fifty-eight (36.5%) women reported alcohol consumption, with 7 (12%) consuming greater than 14 drinks per week. Fifteen (9.4%) women were classified as WHO clinical stage 4. The median CD4 count was 132 (IQR 80–180) cells/µL.

Eleven (7%, 95% CI 3–11) of 159 women were chronic HBV carriers, and these women were followed for a median of 33 (IQR 22–44) months, contributing to a total of 33 person-years of follow-up. An additional 84 (53%, 95% CI 45–61) women had a prior history of HBV infection (HBsAg negative and HBcAb positive). Chronic carriers (HBsAg positive) were more likely to report consuming >14 alcoholic drinks per week (OR 7.68, 95% CI 1.18–49.8) and less likely to report >1 year of transactional sex work at enrollment (OR 0.23, 95% CI 0.06–0.82). Chronic HBV carriers did not differ significantly from other women with respect to age, education level, or reported sexual risk behaviors.

Ten of eleven chronic HBV carriers were infected with HBV genotype A1 (Table 1). One woman (participant 1) did not have baseline HBV DNA levels sufficient for genotypic testing. No baseline lamivudine resistance mutations were detected. Six (55%) of 11 chronic HBV carriers were HBeAg positive at baseline. One participant achieved HBeAg seroconversion after 87 days of ART. Undetectable HBV DNA levels were attained in 9 (82%) women after a median of 88 (IQR 70–322) days. Only one woman (participant 7) relapsed after initially achieving undetectable HBV DNA. Of the five (45%) women who had detectable HBV DNA after 3 months of follow-up, one developed HBV resistance to lamivudine for an incidence among HBV carriers of 3 per 100 person-years.

**Table 1:** Clinical characteristics and treatment outcomes in HIV-1/HBV co-infected Kenyan women.

Participant	Hepatitis BGenotype	HBeAg	HBV DNA Level(IU/mL)	Days to HBV DNA Suppression^a^	Resistance Mutation	Total follow-uptime (months)	Maximum ALT^b^(U/L)	Mean Adherence (percent)^c^
1	Quantity n/s^d^	Negative	350	88	Below limit^e^	33	36	100
2	A1	Negative	1,700	86	Below limit	37	34	92
3	A1	Negative	5,400	28	Below limit	71	20	98
4	A1	Negative	32,000	85	Below limit	38	11	98
5	A1	Negative	63,000	28	Below limit	10	11	100
6	A1	Positive^f^	2,700,000	87	Below limit	11	54	98
7^g^	A1	Positive	4,600,000	392	None	30	136	79
8	A1	Positive	290,000,000	262	None	51	14	97
9	A1	Positive	300,000,000	298	None^h^	17	51	95
10^i^	A1	Positive	490,000,000	Never suppressed	M204I	73	20	97
11^j^	A1	Positive	1,800,000,000	Never suppressed	None	27	14	88

HBeAg, hepatitis B e antigen. HBV, hepatitis B virus.

a HBV DNA Suppression defined as ≤100 IU/mL.

b Upper limit of normal for ALT assay is 17 U/L.

c Mean adherence calculated from pill count across all visits. If pill count unavailable, used patient recall of pills taken.

d Quantity n/s, quantity not sufficient for baseline genotype analysis.

e If HBV DNA <100 IU/mL after 3 months, samples were below the limit for detection and sequencing was not performed.

f This woman seroconverted for HBeAg at day 87 of ART.

g This woman developed ALT elevations and had to discontinue ART at day 58; she resumed at day 334 and initially suppressed HBV DNA at day 392; she was noted to have poor adherence and HBV DNA relapse at day 456.

h Had baseline mutation V173L.

i This woman developed the M204I lamivudine resistance mutation at day 392 of ART; the lowest HBV DNA level achieved was 1100 IU/mL at day 1320 of ART.

j This woman was noted to have poor adherence (88%); the lowest HBV DNA level achieved was 44,000 IU/mL at day 351 of ART.

Overall, seven (64%) women had ALT elevations above the upper limit of normal (ULN: 17 U/L). One woman (participant 7) developed a severe ALT elevation (>5× ULN) as defined by the AIDS Clinical Trials Group and discontinued ART for a portion of follow-up [19]. Compared to women with HIV-1 infection alone, risk of ALT elevation was greater among HIV-1/HBV co-infected women (HR 2.37; 95% CI 1.07–5.24), which was similar after adjusting for alcohol use (HR 2.27; 95% CI 0.97, 5.31). There were no associations between HBV co-infection and time to death, time to CD4 count >350, or HIV-1 suppression.

## Discussion

This study of HIV-1-seropositive Kenyan women demonstrated a 7% prevalence of chronic HBV infection, consistent with the 6–12% found in previous Kenyan studies [7], [16]. Over a median of 33 months, 8 (73%) of 11 chronic HBV carriers demonstrated a durable response to lamivudine monotherapy by maintaining suppression of HBV DNA. The three women who did not maintain suppression (participants 7, 10, and 11, Table 1) all had high HBV DNA levels and were HBeAg positive. Two of these women were noted to have poor adherence, and the third developed lamivudine resistance to HBV.

The development of lamivudine resistance in this cohort was substantially lower than the 40%–50% reported at two years in studies from Western cohorts of HIV-1/HBV co-infected individuals taking lamivudine as the only drug active against HBV [9], [20], [21]. Data from sub-Saharan Africa have suggested potentially lower levels of lamivudine resistance (<15%), but the few published studies have had retrospective or cross-sectional designs and smaller sample size [12], [15]. The discrepancy between African and Western cohorts could be related to differences in the timing of HBV infection in relation to HIV-1 infection or to study design. Studies have also investigated whether the different HBV genotypes account for differences in clinical outcomes including development of lamivudine resistance mutations [22].

HBV has been classified into 8 genotypes, A-H, with genotypes A, D, and E predominant in sub-Saharan Africa [22], [23]. An early study of patients in Germany suggested a 20-fold increased risk of lamivudine resistance mutations in a small cohort (n = 26) with HBV serotype adw compared to ayw (corresponding to genotypes A and D, respectively [24]. However, prolonged clinical observation showed that this difference did not persist beyond one year of therapy [25], [26]. Several subsequent studies comparing genotypes from Asia [27]–[29]), Australia [30], and North America [31] have failed to demonstrate a clear role of genotype in determining clinical outcomes. Problems with these studies include small sample size [28], cross-sectional design [31], and pooling of patients from different ethnicities [30], [31]. However, recent large-scale prospective studies from China show higher rates of YMDD mutations in genotype B compared to C [32], [33]. In summary, the available literature provides a somewhat conflicting picture, and questions persist as to whether HBV genotypes influence response to therapy and lamivudine resistance.

A recent prospective cohort study of HIV-1/HBV co-infected adults in Nairobi, Kenya found a similarly low incidence of lamivudine resistance to the present study (7/100 person-years) [16]. Interestingly, in both our study and the Nairobi study, only HBV genotype A1 was identified. While our cohort in Mombasa had a lower absolute number of chronic HBV carriers (11/159 [6.9%] versus 27/389 [6.9%]), the participants contributed substantially longer periods of follow-up (median 33 versus 18 months). The present cohort also differed from the Nairobi cohort by having higher baseline HBV DNA levels (73% versus 33% with HBV DNA >10,000 IU/mL) and HBeAg-positivity (55% versus 11%), both of which have been associated with increased risk of lamivudine resistance [20], [34], [35]. With such different markers of HBV infection but similarly low rates of lamivudine resistance, these studies suggest that HBV treatment outcomes to lamivudine monotherapy in the region may be distinct from what has been published elsewhere.

The unique strength of our study was the extended period of follow-up of this prospective cohort, which enabled us to ascertain virologic outcomes over a substantially longer period than previous studies [9], [12], [16], [34]. In addition, we included all individuals with HIV-1/HBV co-infection on ART in an intent-to-treat analysis. In contrast, some studies in both Western and African cohorts have included only the subset of participants who have detectable plasma HBV DNA during follow-up [12], [20]. This approach may overestimate lamivudine resistance by excluding participants with sustained undetectable plasma HBV DNA levels.

The main limitation of this study was the relatively small number of women with chronic HBV infection. Although loss to follow-up in this study would not affect baseline prevalence estimates of HIV-1/HBV co-infection, it could limit evaluation of outcomes. In addition, this exploratory study included multiple statistical comparisons, affecting our ability to interpret significant results. Since time on lamivudine is a risk factor for development of lamivudine resistance [9], using the intent-to-treat principle and leaving HIV-1/HBV co-infected individuals who discontinue lamivudine in the analysis could falsely lower our detection of resistance mutations. However, only one of 11 women (participant 7) discontinued lamivudine but remained in follow-up. This study did not include testing for hepatitis C or hepatitis D, both of which are important co-infections in HIV-infected individuals. However, their exclusion should not limit interpretation of the HBV results. Finally, the ADVIA Centaur HBsAg assay used in this study may be limited in its ability to detect certain HBsAg mutants [36], [37]. The significance of this limitation is difficult to assess, as prevalence estimates of HBsAg mutants in sub-Saharan Africa are not widely available. A recent study evaluating 11,221 HBV sequences of genotypes A-H demonstrated a low prevalence of HBsAg variants in genotype A viruses (54/1,524, or 3.6%) [38]. Failure to detect HBsAg mutants in our present study could have resulted in a small number of missed cases of chronic HBV infection.

As increased access to ART increases life expectancy of HIV-1/HBV co-infected individuals in sub-Saharan Africa, HBV is expected to be an important cause of morbidity and mortality in these individuals [2], [6], [7]. As of 2010, WHO recommends HBV screening before initiating ART, and using ART regimens containing tenofovir with either lamivudine or emtricitabine for HIV-1/HBV co-infection [10], [11]. However, in resource limited settings, HBV screening may be unavailable [13]. As a result, many HIV-1/HBV co-infected adults are likely to be exposed to lamivudine monotherapy as part of first or second-line ART regimens. Our study was conducted in a high risk population of women in East Africa, and these results may not be generalizable to men or other regions where different genotypes are encountered. Nonetheless, our results suggest that the incidence of HBV genotypic resistance during lamivudine monotherapy may be lower in some populations. This was also suggested in a recent Canadian study of 47 HBV mono-infected adults on lamivudine for a mean of 32 months. Sustained HBV DNA suppression was observed in 89% of patients, and they concluded that lamivudine monotherapy may be appropriate in some populations [39]. It will be important to study larger cohorts from diverse regions to determine the impact of different ART regimens on the clinical outcomes of HIV-1/HBV co-infection. Expanding access to HBV screening could facilitate rational sequencing of antiretrovirals for these patients, potentially leading to improved long term outcomes.
